# Left Posterior Orbitofrontal Cortex Is Associated With Odor-Induced Autobiographical Memory: An fMRI Study

**DOI:** 10.3389/fpsyg.2018.00687

**Published:** 2018-05-11

**Authors:** Keiko Watanabe, Yuri Masaoka, Mitsuru Kawamura, Masaki Yoshida, Nobuyoshi Koiwa, Akira Yoshikawa, Satomi Kubota, Masahiro Ida, Kenjiro Ono, Masahiko Izumizaki

**Affiliations:** ^1^Department of Physiology, Showa University School of Medicine, Tokyo, Japan; ^2^Department of Neurology, Showa University School of Medicine, Tokyo, Japan; ^3^Department of Ophthalmology, The Jikei University School of Medicine, Tokyo, Japan; ^4^Human Arts and Sciences Research Center, University of Human Arts and Sciences, Saitama, Japan; ^5^Department of Radiology, Stroke Center, Ebara Tokyo Hospital, Tokyo, Japan

**Keywords:** olfaction, autobiographical memory, posterior orbitofrontal cortex, arousal, vividness, breathing, anterior cingulate cortex (ACC)

## Abstract

Autobiographical odor memory (AM-odor) accompanied by a sense of realism of a specific memory elicits strong emotions. AM-odor differs from memory triggered by other sensory modalities, possibly because olfaction involves a unique sensory process. Here, we examined the orbitofrontal cortex (OFC), using functional magnetic resonance imaging (fMRI) to determine which OFC subregions are related to AM-odor. Both AM-odor and a control odor successively increased subjective ratings of comfortableness and pleasantness. Importantly, AM-odor also increased arousal levels and the vividness of memories, and was associated with a deep and slow breathing pattern. fMRI analysis indicated robust activation in the left posterior OFC (L-POFC). Connectivity between the POFC and whole brain regions was estimated using psychophysiological interaction analysis (PPI). We detected several trends in connectivity between L-POFC and bilateral precuneus, bilateral rostral dorsal anterior cingulate cortex (rdACC), and left parahippocampus, which will be useful for targeting our hypotheses for future investigations. The slow breathing observed in AM-odor was correlated with rdACC activation. Odor associated with emotionally significant autobiographical memories was accompanied by slow and deep breathing, possibly involving rdACC processing.

## Introduction

A number of studies have reported that a feeling of “going back in time” is experienced more strongly for odor-evoked episodic (autobiographical) memories than for those elicited by verbal or visual cues (Herz and Cupchik, 1992; Herz, 2004). Unlike other types of autobiographical memory, autobiographical odor memory (AM-odor) often accompanied by a sense of realism of a specific memory that elicits strong emotions. These features of olfaction-related memories might be the result of the unique sensory process that distinguishes olfaction from other sensory modalities. Olfaction affects respiration activity, and olfactory information ascend directly to olfactory-related limbic structures, including the piriform cortex (Pir), entorhinal cortex (ENT), amygdala (AMG), hippocampus (HI), and orbitofrontal cortex (OFC) bypassing the thalamus. These areas overlap with areas related to emotion and memory. The involvement of these brain areas in human olfaction and emotion has been confirmed by a number of neuroimaging studies (Zatore et al., 1992; Sobel et al., 1998; Rolls, 2004; Masaoka et al., 2012a, 2014). In addition, Herz (2004) reported that, compared with memories evoked by other sensory cues, AMG and HI activation were specifically activated during AM-odor. Moreover, another study reported strong activation in the HI when recalling autographical memory associated with a specific odor (Masaoka et al., 2012a).

Odor information ascends directly to the olfactory limbic cortex and induces the retrieval of emotional and episodic memory. The ENT and the AMG outputs finally converge on the OFC, where higher-order processing subsequently takes place, including smell identification and emotional labeling (Rolls, 2001).

The OFC plays a role in the recognition and naming of the odor, and may also provide signals relating to the internal and emotional environment. Although olfactory recognition has been reported to activate the OFC, the roles of specific OFC sub-regions in relation to AM-odor have not yet been clarified. In addition, because odor-induced autographical memory involves positive emotional significance for personal episodic and spatial memory, AM-odor should activate not only the olfactory limbic regions AMG, HI and OFC, but also widespread areas of the brain. Thus, activation of the limbic areas and OFC may reflect increased connectivity with other cortical areas.

Our study used functional magnetic resonance imaging (fMRI) and respiration measurement during AM-odor to examine two main questions: first, we investigated which sub-regions of the OFC were associated with AM-odor, and which areas were activated during this form of processing. To examine this, connectivity analysis was performed between the OFC and the whole brain. Second, we sought to investigate the relationships between cortical areas and respiratory activities. Stimulation of the AMG is known to increase respiratory frequency during states of fear and anxiety (Masaoka and Homma, 2004). However, AM-odor has been reported to specifically decrease breathing frequency, and is associated with a deep and slow respiratory pattern (Masaoka et al., 2012b), even though the AMG is thought to be involved in olfactory processing. Thus, we hypothesized that the OFC and/or other cortical regions might contribute to slowing the rate of breathing, potentially involving an inhibitory system for respiration during emotionally significant states.

## Materials and Methods

### The AM-Odor Pretest – Selection of Odors for the Experiment

A total of 17 subjects (all male) were selected from 30 volunteers (age range: 31–50 years) on the basis of a pretest interview. Details of the pretest were reported in a previous study (Masaoka et al., 2012b). Briefly, we interviewed 30 volunteers to determine the odor stimuli used in the experiment. All 30 volunteers were asked to describe their memory, and rate the level of emotion they felt in response to the odor by answering the following questions (Masaoka et al., 2012b):

(1) Have you experienced a certain odor that elicits a specific memory associated with a person, place, or event?
(2) Identify the name of the odor that triggers your specific memory.
(3) Write a brief description of the memory.
(4) Rate the pleasantness felt when recalling the memory from the odor and the memory context (1 = very unpleasant; 5 = very pleasant).
(5) Rate the vividness of the memory context (1 = not at all strong; 5 = extremely strong).
(6) How strong was the feeling of being taken back in time to the occurrence of the event? (1 = not at all strong; 5 = extremely strong)
(7) How emotionally intense was your memory related to the odor? (1 = not at all strong; 5 = extremely strong)

A group of 30 subjects identified the odor which elicited a specific memory. The odors were tatami, pyrethrum coil, osmanthus, rose, cypress, incense stick, antiseptic solution, camphor, baby powder, citrus and bonfire. Of these, three (tatami, osmanthus, baby powder) were common AM odors to this group of subjects. Of 20 subjects, six chose tatami, seven chose osmanthus, and seven chose baby powder. These 20 subjects were then tested with the above questionnaire (from above questions 2–7) while being presented with a control odor, *β*-phenyl ethyl alcohol (PEA), which we used in previous studies (Masaoka et al., 2012b). In our previous study (Masaoka et al., 2012b), we used two control odors; PEA and chamomile, which had characteristics of a normal odor that was not induced memory retrieval. These two odors were not significantly different in their emotional attributes (pleasantness, comfortable level, arousal level, and familiarity) or respiratory responses (Masaoka et al., 2012b). To optimize the scanning time and to avoid mixing odors in the odor delivery valve in the scanner, we used one control odor in the current study. Of the 20 subjects, 17 reported that the control odor did not induce a memory of the past, while three reported that the control odor did. In addition, we tested whether subjects felt pleasantness in response to the control and AM odors (control odor, 3.7 ± 0.8, AM-odor, 3.9 ± 0.75, *P* > 0.05), vividness of the memory context (control odor, 1.2 ± 0.2, AM-odor, 4.5 ± 0.21, *P* < 0.05), the feeling of being taken back in time (control odor, 1.4 ± 0.3, AM-odor, 4.3 ± 0.18, *P* < 0.05) and the emotional intensity (control odor, 3.2 ± 1.1, Am-odor, 4.2 ± 1.5, *P* < 0.05). These 17 subjects participated in the fMRI study. Further details of the pretest for AM-odor can be found in our previous study (Masaoka et al., 2012b).

After the fMRI scan, we instructed participants to fill out the following questionnaire (Masaoka et al., 2012b) for both the control odor and AM-odor:

(1) How aroused were you during retrieval of the memory related to the odor? (1 = very calm; 5 = very aroused).
(2) How comfortable was your memory related to the odor? (1 = not at all comfortable; 5 = very comfortable).
(3) Rate the degree of pleasantness you felt when recalling the memory from the odor and the memory context (1 = very unpleasant; 5 = very pleasant).
(4) How vivid was the memory (1 = very ambiguous; 5 = extremely vivid).

### Participants

Seventeen right-handed healthy volunteers selected by pretesting (age range: 31–48 years, mean: 36 ± 5.6 years) participated in fMRI study. All participants had a normal sense of smell as determined by T&T olfactometer (Takasago Industry, Tokyo, Japan). All participants were free from allergies and had normal respiratory function.

This study was reviewed and approved by the Ethics Committee of Showa University School of Medicine. Written informed consent was given by all participants prior to the experiment.

### fMRI Design for Olfaction

The fMRI testing was divided into two sessions: (1) periods of AM-odor interspersed with unscented air (baseline), and (2) periods of control odor (rose) interspersed with unscented air (baseline). Each session comprised five unscented and five scented 30-s blocks. We used a design with 30-s presentations of scented and unscented air to minimize adaptation to the olfactory stimuli (Ekman et al., 1967). In addition, we used this 30-s block design because breathing changes were previously observed during AM-odor, returning to a normal breathing pattern in the 30 s-unscented phase (Masaoka et al., 2012b). Session types (control odor session or AM-odor session) were performed in randomized order on the same day, and at the same time (PM6:00-7:00). To control for sleep, we asked all subjects to sleep before 11:00 P.M, and to sleep for 7 h on the day before the experiment (6.55 ± 1.2 h).

Participants were instructed to breathe normally through a nose mask designed for delivering odors and measuring respiratory flow. Further details of this design are described in a previous study (Masaoka et al., 2014). Odorants were administered using a custom-designed, MRI-compatible apparatus (Acro System, Chiba, Japan). Participants wore a nose mask (ComfortGel Blue Nasal Mask 1070038, medium size, Phillips Respironics, Murrysville, PA, United States) fitted with a one-way valve apparatus to ensure inspiration of air from the olfactory stimulator and expiration out of the system. A piezoelectric pressure transducer was connected to the respiratory pressure sensor in the nose mask by urethane tube. The pressure signal measured inspiratory and expiratory flow, which was converted from an analog to a digital signal within a control box and stored together with cardiac output (Bio Pac, LA system, Japan) in LabChart through PowerLab (ML846, AD Instruments, Aichi, Japan) (Masaoka et al., 2014). In addition, we measured end-tidal CO_2_ in seven of 17 subjects throughout the experiment (O_2_ and CO_2_ Analyzer, Acro System, Chiba, Japan).

### fMRI Data Collection and Analysis

The MRI scanning was performed at Ebara Hospital (Tokyo, Japan) using a 3 Tesla MAGNETOM A Trio Tim scanner (Siemens, Erlangen, Germany) with a 32-channel head coil. Functional imaging was acquired with multiband (MB) accelerated gradient-echo echo-planar imaging that excited four slices simultaneously (MB = 4) to increase temporal resolution. The fMRI time-series consisted of 330 whole-brain volumes/session, each comprising 39 axial slices (Matrix: 80 × 80; TR: 1 s; TE: 27 ms; FOV: 16–22 cm, thickness: 2.5 mm; Flip angle: 90°). Magnetic field inhomogeneities in brain regions close to bone and air-filled sinuses led to a reduction of the signal-to-noise ratio and signal loss (O’Doherty et al., 2002). To increase the signal-to-noise ratio and reduce signal loss, an echo time (TE) of 27 ms was employed in this study (Gorno-Tempini et al., 2002). Lower TE (27 ms) makes it possible to detect activations without the effects of susceptibility artifacts (Gorno-Tempini et al., 2002). Anatomical MRI was also acquired using 3D-magnetization-prepared rapid-acquisition-by-gradient-echo T1-weighted sagittal sections.

Statistical analysis of fMRI data was performed using statistical parametric mapping (SPM12) software (Wellcome Department of Cognitive Neurology, London, United Kingdom) implemented in MATLAB (R2013B; Math Works Inc., Natrick, MA, United States) on a computer running Mac OS X Yosemite. Image pre-processing included rigid-body motion correction (realignment), co-registration of functional and structural images, normalization to standard space, physiological noise correction (SPM8, Drifter Toolbox) and spatial smoothing using a 6 mm FWHM Gaussian filter (Ashburner and Friston, 2003). For each session in each participant, fMRI time-series were modeled using a single regressor encoding the blocked on-off periods of the experiment, convolved with a canonical hemodynamic response function.

Single participant SPM contrast images (first-level) were estimated by comparing the AM-odor and control-odor conditions with the baseline (no-odor) condition. The resulting first-level contrast images were then subjected to second-level analysis. Paired *t*-tests were performed for the *AM-odor > control-odor* contrast across whole brain and regions of interest (ROIs) analyses. The ROIs were defined anatomically using the Automated Anatomical Labeling (AAL) atlas (Tzourio-Mazoyer et al., 2002) based on previous imaging studies of olfaction (Zatore et al., 1992; Sobel et al., 1998; Rolls, 2004; Masaoka et al., 2012a) and our previous olfaction imaging study (Masaoka et al., 2014), and included bilateral olfactory areas, the parahippocampus, AMG, HI, insula (Insula), frontal medial (Frontal_Med), middle (Frontal_Mid), inferior (Front_Inf) and superior (Front_Sup), orbitofrontal (OFC), and superior/middle temporal pole (Temporal_Pole_Sup). Voxel-wise statistical threshold maps were constructed for inference using statistical thresholds corrected for multiple comparisons (cluster-based family wise error corrected *P* < 0.05) based on Monte Carlo simulations, as implemented using the AlphaSim toolbox (AFNI; National Institutes of Mental Health, Bethesda, MD, United States). Null cluster-extent probability distributions were estimated based on 1000 permutations of simulated Gaussian noise with a spatial smoothness (FWHM) of 9.6 mm (estimated using SPM 12) within the analysis mask. Contiguous clusters in the noise field were defined by edge or diagonally connected voxels meeting a cluster forming threshold of 186 voxels (*P* < 0.01). The permutation method enables control of false positives, and is a non-parametric method for making weak assumptions about the data, enabling testing of the null hypothesis by simply permuting observations (Winkler et al., 2014).

The context of AM-odor might modulate the functional connectivity between the seed region and other brain regions. Thus, we conducted psychophysiological interaction analysis (PPI) (Friston et al., 1997) to explore functional connectivity. PPI computes functional connectivity between the time series of a seed voxel of interest (VOI) and the time series of all other voxels, aims to identify regions in which activity depends on an interaction between task (AM-odor and control odor) and physiological factors (the time course of regions of interest). PPI analysis was carried out for each subject with SPM 12, and the resulting images of contrast estimates were entered into a random effects group analysis (one-sample t test for the VOI, and a multiple regression model for whole brains to examine the conjunctive PPIs of whole brain regions). The statistical significance was set at voxel-wise *P* < 0.01 uncorrected and an extent threshold of cluster-wise *P* < 0.05 uncorrected.

Our second aim concerned the hypothesized relationship between respiratory pattern changes and cortical activation. The respiratory change was represented as the computed difference between baseline and during odor conditions (RR*dif*). We examined associations between these respiration changes and neural responses associated with *AM-odor > control-odor* contrast. The results were initially small-volume corrected (SVC)(FEW_SV C_ < 0.05) (*t* > 3.85, cluster > 16) with a 6-mm sphere radius at the peak coordinate of MPFC/rdACC cluster based on the hypothesis that the MPFC involved physiological and autonomic changes (Wagner et al., 2009).

All areas of activation were overlaid on the template image (ch2bet.imag) implemented within MRIcron^1^.

### Statistics for Cardio-Respiratory Measurements and Subjective Ratings

Respiratory rate (RR), heart rate and end-tidal CO_2_ (ETCO_2_) were measured during baseline (five unscented 30-s blocks) and each olfaction condition (five scented 30-s blocks). Changes of RR, heart rate and ETCO_2_ were tested using one-way analysis of variance (repeated ANOVA), and *post hoc* testing was performed with Bonferroni correction. Subjective ratings pre- and post-test for arousal level, comfortableness, pleasantness, and vividness of the memory were compared between the AM-odor and control-odor conditions using a Wilcoxon signed-rank test.

## Results

### RR, Heart Rate and ETCO_2_ During fMRI Scanning

**FIGURE 1:**
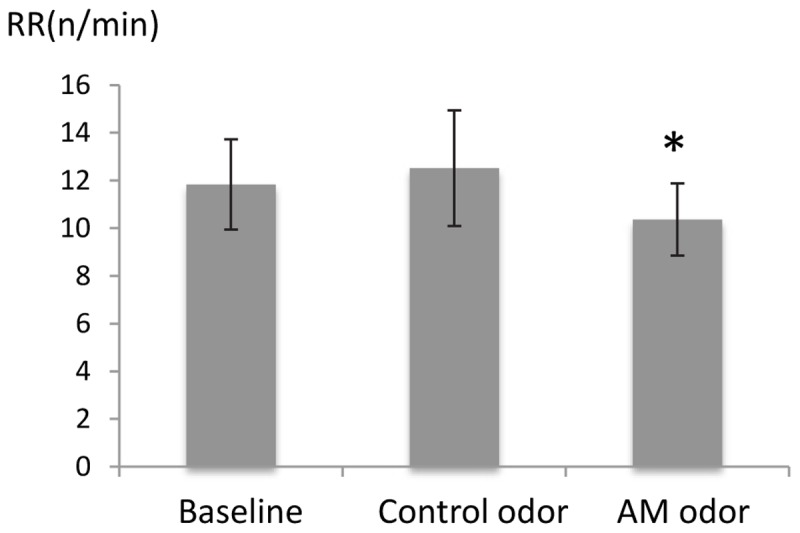
Comparison of respiratory rate (RR) between control odor and autobiographical odor memory (AM-odor). ^∗^*P* < 0.05.

There was a significant difference in RR (*F* = [2,48] = 3.02, *P* < 0.05), and *post hoc* tests showed that RR was significantly reduced during AM-odor stimuli (*P* < 0.05; **Figure 1**, indicated in minutes). There was no difference in heart rate (indicating mean ± standard error, baseline, 68.8 ± 1.54, control odor, 68.9 ± 1.54, AM-odor, 68.7 ± 1.49, *F*[2,48] = 0.006, *P* > 0.05), and ETCO_2_ (baseline, 4.72 ± 0.08, control odor, 4.68 ± 0.04, AM-odor, 4.7 ± 0.03, *F*[2,18] = 0.06, *P* > 0.05) throughout baseline for both olfactory stimuli.

### Subjective Ratings for AM-Odor

**FIGURE 2:**
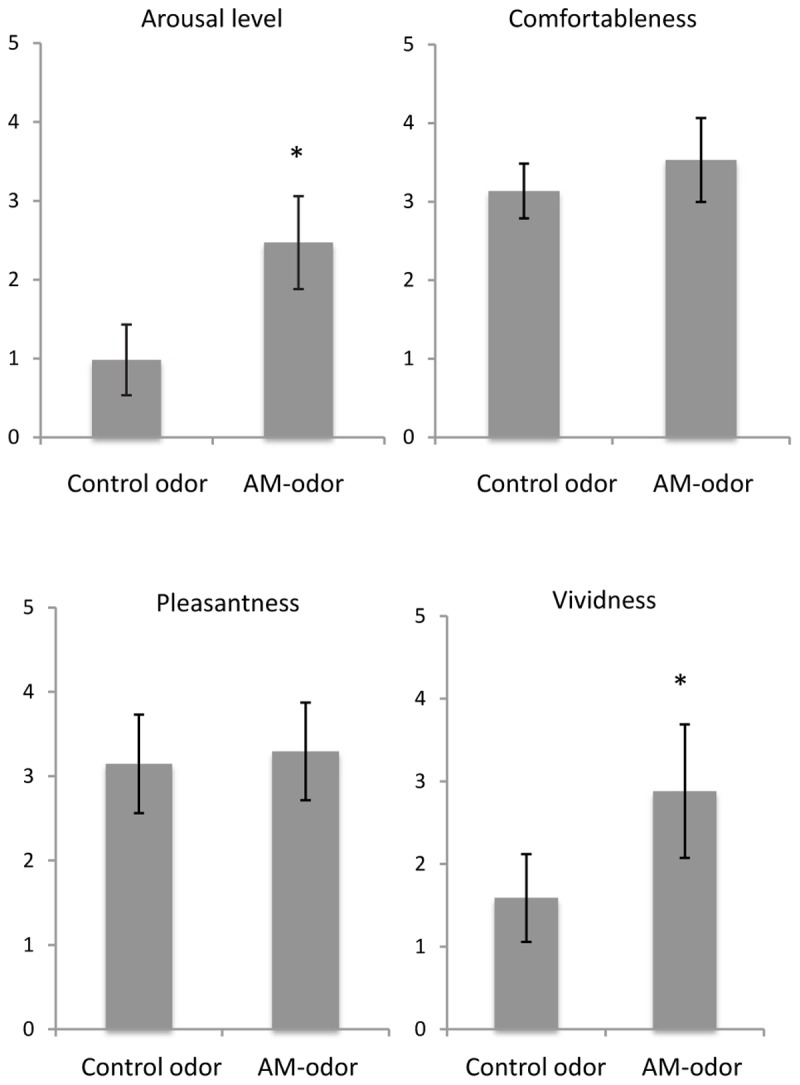
Comparisons of subjective scales between control odor and autobiographical odor memory (AM-odor). ^∗^*P* < 0.001.

**Figure 2** shows a comparison of subjective ratings between control odor and AM-odor. We found that arousal level was significantly higher in the AM-odor session than the control-odor session (AM-odor: 2.47 ± 0.58, control odor: 0.98 ± 0.4; *t* = 8.6, df = 16, *P* < 0.001), and that memories were more vivid (AM-odor: 2.88 ± 0.8, control odor: 1.5 ± 0.53; t = 6.9, df = 16, *P* < 0.001). In contrast, comfortableness (AM-odor: 3.5 ± 0.53, control odor: 3.1 ± 0.3) and pleasantness (AM-odor: 3.3 ± 0.6, control odor: 3.1 ± 0.6) did not differ between the two sessions (comfortableness, *t* = 1.8, df = 16, *P* = 0.07; pleasantness, *t* = 0.73, df = 16, *P* = 0.47). Subjective ratings indicated that both odors successively induced comfortable and pleasant feelings, but only the AM-odor increased arousal level and induced more vivid memory, both of which involve specific autographical memory.

Three examples of the episodic memories reported by subjects are presented below:

Example 1: One participant remembered being in a living room after taking a bath with his mother. His mother was trying to catch him as he ran away while wearing pajamas. She caught him and put baby powder on his shoulder.
Example 2: One participant remembered being in the tatami room at his grandmother’s home. The participant remembers that their grandmother was always gentle, and they played a game on the tatami mat.
Example 3: One participant remembered being at a playground near the home he was raised in. He often played outside until late, and his mother always called him for dinner time. On the way home, he smelled osmanthus flowers, his favorite.

### Brain Activation

**FIGURE 3:**
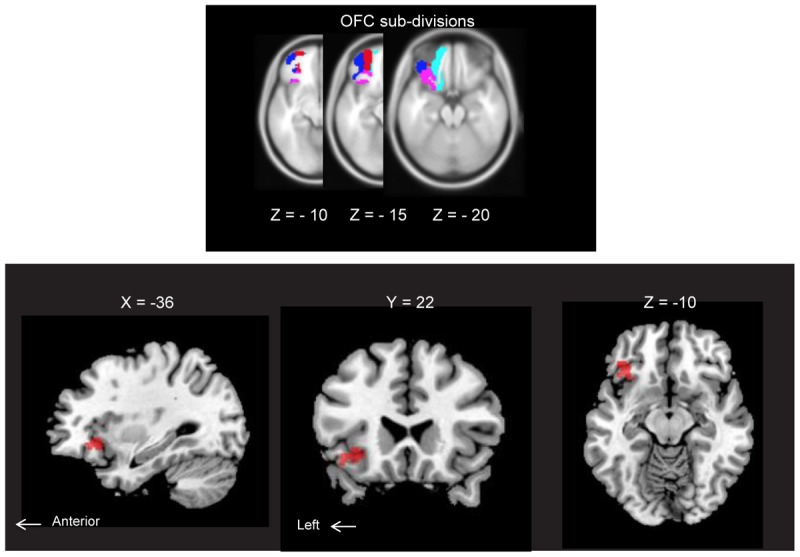
**(Upper)** Horizontal section of orbitofrontal cortex (OFC) subdivisions: lateral OFC (blue), medial OFC (cyan), anterior OFC (red) and posterior OFC (pink). Each structure was extracted from an image of neuromorphometrics labels in SPM12 (Wellcome Department of Cognitive Neurology, London, United Kingdom) and overlaid on MNI space. All subdivisions are indicated for the left hemisphere only. **(Lower)** Brain regions showing AM-odor specific responses (the *AM-odor > control odor* contrast). Results corresponded to FWE corrected *P* < 0.05 (MNI coordinates: *X* = –36, *Y* = 22, *Z* = –10, *T* = 3.53, cluster size = 251). These regions also survived at a whole-brain threshold of *P* < 0.001, uncorrected (cluster extension 10 voxels).

ROI analysis of the *AM-odor > control*-*odor* contrast revealed activation in the left posterior OFC (POFC). Results corresponded to family-wise error (FWE)-corrected *P* < 0.05 (MNI coordinates: *x* = -36, *y* = 22, *z* = -10, SPMz = 3.53, cluster size = 251) (**Figure 3**). This area also survived for whole-brain level, *P* < 0.001, uncorrected.

Preservation of the signal in the basal frontal region is shown in the Supplementary Figure 1. Exploratory analyses across the whole brain did not reveal any additional regions of significance. Activations in other areas with thresholds of *P* < 0.05, *P* < 0.01, and *P* < 0.001 uncorrected for whole brain analysis together with POFC activations were included as exploratory results in Supplementary Figure 2.

### Psychophysiological Interaction Analysis

We set the left POFC as the VOI, and conducted PPI analysis to examine the functional connectivity between the left POFC and whole brain regions (Supplementary Table 1). Because PPI analyses did not reveal significant connectivity effects, we consider these findings to reflect exploratory trends.

PPI of the left POFC seed had trends to increase connectivity between the left POFC and bilateral precuneus, left middle pre-frontal cortex (MPFC)/rostral dorsal anterior cingulate cortex (rdACC), and left parahippocampus (voxel-wise *P* < 0.01 uncorrected with an extent threshold of cluster-wise *P* < 0.05 uncorrected). We found trends toward increased connectivity between the left POFC. The left POFC seed showed trends toward negative coactivation with bilateral anterior cingulate, left AMG, left anterior insula and left MPFC/ventromedial PFC (vm-PFC) (voxel-wise *P* < 0.01 uncorrected with an extent threshold of cluster-wise *P* < 0.05 uncorrected).

### Correlations With Subjective Scales and Respiratory Rate

**FIGURE 4:**
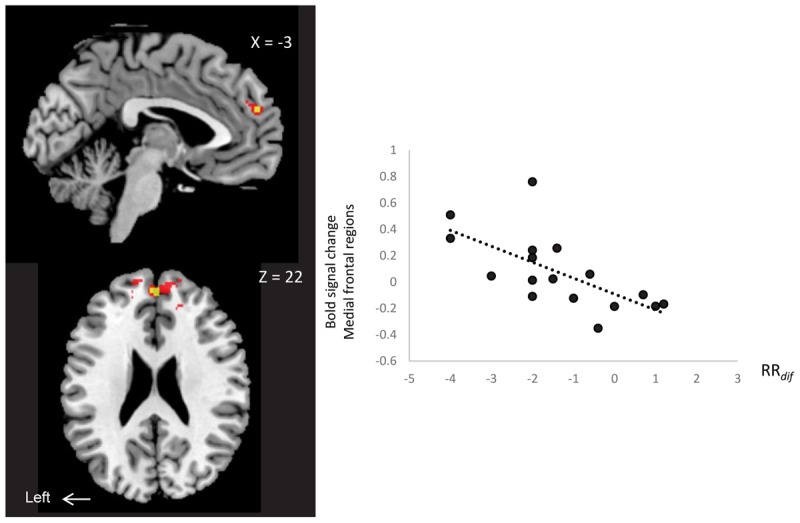
A negative correlation between MPFC/rdACC activity (*x* = 0, *y* = 52, *z* = 22) (*AM-odor > control odor* ) and RR_dif._ Scatter plot representation is shown on the right. *P* < 0.01 (red) for display purposes. Results in yellow correspond to FWE-corrected small volume correction *P* < 0.05.

We examined neural correlates of RR*dif* (baseline-AM-odor state), and subjective scales (arousal level, comfortableness, pleasantness and vividness) with whole brain regions. There was a significantly negative correlation between MPFC/rdACC and RR_dif_ (*x* = 0, *y* = 52, *z* = 22: *t* = 5.25, 16 voxels; FWE_SV C_, *P* < 0.05) (**Figure 4**). Scatter plot representation was indicated in **Figure 4** right (*P* = -0.67, *P* = 0.004). This effect was observed in the contrast of *AM-odor > control odor*. No association was found between subjective scales and brain regions. This area correlated with RR*dif*, and also survived whole brain uncorrected analysis (*P* < 0.001).

## Discussion

### POFC Activation and Connectivity With Other Brain Regions

The present study revealed that the left POFC was activated by odor stimuli associated with autobiographical memory, which might be associated with overall increased arousal levels and vividness of the memory. The POFC is characterized by strong connections with the AMG, midline thalamus, insula, and temporal pole (Mesulam and Mufson, 1982), and receives direct olfactory connections from the Pir (Price et al., 1991). A number of fMRI studies have reported that the POFC is relatively strongly activated in response to pleasant odor stimuli (Gottfried et al., 2002a), attractive human faces (Aharon et al., 2001), and increased arousal during a gambling task (Breiter et al., 2001). Increased arousal and vividness of odor-induced memory in this study may be linked to activation of the POFC involving processing of reward and positive incentive value (Diekhof et al., 2011).

Our PPI analyses did not reveal any significant connectivity effects. This finding may reflect a lack of statistical power relative to investigating the main task effects, which is an established limitation of PPI analyses (O’Reilly et al., 2012). However, there were several interesting trends in the data, which may inform more targeted hypotheses for investigation in future work, including increasing connectivity between the POFC and bilateral precuneus, and between rdACC and the left parahippocampus (see Supplementary Materials for further details), possibly including areas related to spatial and episodic memory (precuneus; Gilboa et al., 2004), memory retrieval (parahippocampus; Steinvorth et al., 2006) and memory and self reflection (medial prefrontal area; Northoff et al., 2006).

### Slow Breathing and rdACC

The results indicated a link between rdACC and respiratory response. The slow breathing observed in AM-odor was negatively correlated with rdACC activation, meaning that subjects with slow breathing tend to increased activation in rdACC.

A recent neuroimaging study elucidated the relationship between physiological response and brain activity. Attention to breath as a basic mindfulness practice down-regulates AMG activation and increases dorso-medial PFC activation (Doll et al., 2016), suggesting that this effect is independent of breathing frequency, but related to attention.

In contrast, the current study suggests that slow breathing by AM-odor might contribute to emotional state (Masaoka et al., 2012b). A large sniffing volume contributes to olfactory mental imagery (Bensafi et al., 2005). This breathing change might be associated with rdACC activation, which is directly linked to the periaqueductal gray (PAG) and dorsal raphe nucleus involved in autonomic changes (Wagner et al., 2009). The PAG connects to the parabrachial nucleus, which plays a role in behavioral changes of respiration, connecting respiratory rhythm generators in the brainstem (Martelli et al., 2013). The details of the interactions between breathing pattern changes in AM-odor and respiratory rhythm generators in the brainstem are currently unclear. Thus, the precise link between the higher cortical areas and brainstem areas in slower breathing by odor-induced autobiographical memory should be investigated in further research.

It is important to note that two respiratory activity factors affecting BOLD signals were removed in this study. First, we observed constant ET_CO2_ levels throughout scanning, indicating that subjects were not hypo or hyperventilating without decrease/increase of arterial CO_2_. Thus, the BOLD signal was not likely to be affected by deep breathing. Second, physiological respiration-related noise was removed in preprocessing, and artifacts related to respiratory movement were minimized in the BOLD signal.

It should also be noted that other subjective scales such as comfortableness and arousal level increased during AM-odor, but these scores were not correlated with specific brain regions. This pattern of results may reflect an increase in overall scores during the AM-odor state, without individual differences in this increase. Future studies with a larger number of subjects should be conducted using another type of scale: for example, a visual analog rather than a categorical scale.

### Laterality

In the current study, we found that activation in the left POFC was specific to AM-odor. Laterality of the OFC for pleasant olfactory processing has been reported in a number of studies. Odor-induced pleasant emotions activated right OFC (Gottfried et al., 2002b) and Rolls et al. (2003) reported that unpleasant odor activated the left OFC. However, Royet et al. (2003) reported that left OFC activation was dominant for pleasant odors, suggesting a role for this area in conscious assessment of the emotional quality of odors. A meta-analysis of the functional neuroanatomy for autobiographical memory indicated a core neural network of left-lateralized regions, including the medial and ventrolateral prefrontal cortices (Svoboda et al., 2006). Thus, autographical memory and related processes, including semantics, visual imagery, and emotional arousal, might be associated with the left hemisphere. It should be noted that the subjects in this study were all right-handed, although handedness of subjects and laterality of brain activation during memory retrieval and emotional arousal are unknown variables.

### Primary Olfactory Cortex

The POFC activation we observed might have been associated with emotional/cognitive higher brain function. However, primary olfactory areas including the Pir, ENT, and AMG were not significantly activated at the group level. Individual analysis showed activation in the olfactory limbic regions including primary olfactory areas (*P* < 0.05 FWE-corrected) in the subjects (see Supplementary Figure 3 and Supplementary Table 2), but some subjects did not exhibit significant activation.

This lack of significant primary olfactory fMRI activation at group level might be due to rapid habituation of these regions to olfactory stimuli (Sobel et al., 2001). Repeated presentation of the same odorant leads to decreased neural responses in the Pir, ENT, and AMG, and, in turn, a decreased blood-oxygen-level dependent (BOLD) response. The habituation may also vary from person to person. Although the primary olfactory areas habituate very quickly in some subjects, downstream secondary areas such as the OFC continue to respond to recurrent odor presentation (Poellinger et al., 2001). The distinct temporal profiles governing primary versus secondary olfactory processing may explain why detection of robust fMRI activation at the group level was restricted to the OFC in the current study.

This characteristic profile of olfactory habituation may play a role in ensuring sensitivity to new odorant stimuli (Masaoka and Homma, 2009). It may be useful for future studies to investigate the possible connection between habituation level and individual emotions, such as anxiety.

### Limitations and Future Directions

The current study involved several limitations that should be considered. First, in a PPI analysis, the psychological context, activity in the seed regions and the interaction between brain areas are all modeled, and tend to lack statistical power because the expected effect size would be much smaller than for an analysis of the main effect of task.

Second, AM-odor was chosen based on a pleasant memory from the past. For ethical reasons, we did not investigate AM-odor associated with unpleasant experiences and situations. For odor presentation, we were only able to use two odors (AM-odor and control odor) to avoid mixing odors in the valve that delivered the odors to participants.

Although we confirmed that the control odor caused pleasant and comfortable feelings, but was not related to autographical memory individuals, it may have induced a range of other phenomena, such as thinking about previous presentations of the odor.

In future research, various types of AM-odors and control odors should be tested with a remodeled olfactory delivery system. In addition, future studies should compare the effects of odors with various characteristics on brain activation (e.g., comparing the effect of novel odor on brain activation vs. the effect of thinking of a previously presented novel odor on brain activation). It would be useful to compare brain activity when autobiographical memory is induced by other sensory modalities. It should be noted that odor perception is known to be influenced by the menstrual cycle in women (Doty and Cameron, 2009). To avoid this potential difficulty in the current study, we only tested male subjects. This difference should be examined in future research.

## Summary

AM-odor increased arousal levels and strength of memories, and was associated with activation of L-POFC. AM-odor was associated with slower breathing, and this slow breathing was associated with rdACC activation. The relationship between slow breathing, AM-odor and higher brain regions is unclear, particularly in terms of its interaction with prefrontal areas. Although the finding was not statistically significant, we observed a trend toward increased connectivity between POFC and rdACC in PPI analysis, which will aid the targeting of hypotheses for future investigations.

The storage of long-term memory in prefrontal regions isolated from hippocampal memory encoding has been confirmed in animal studies (Kitamura et al., 2017). Thus, it may be interesting to investigate whether autographical memory is related to prefrontal regions, or whether memory accompanied with physiological responses (slow breathing) is stored in this region.

Negative emotions such as anxiety and fear have been reported to increase respiratory frequency as part of defensive responses (Davis, 1992), and the amygdala plays a role in behavioral responses. However, the meaning of slow breathing during pleasant emotional experience remains unclear.

Respiratory outputs express opposite responses during the experience of negative and positive emotions. The rdACC and slow breathing observed in the current study may provide insight into the potential inhibitory mechanisms of excessive activation of the amygdala observed in anxiety and panic disorders.

## Ethics Statement

This study was carried out in accordance with the recommendations of ethics committee of Showa University School of Medicine in accordance with the ethical standards as laid down in the 1969 declaration of Helsinki. Written informed consent was obtained from all participants included in the study.

## Author Contributions

KW and YM designed the study, conducted the experiments, analyzed the data, and wrote the initial manuscript. MY, NK, AY, SK, MId, and KO recruited participants, assisted with scanning, and performed pre-processing of fMRI data. All authors discussed the results. YM, MK, and MIz edited the final manuscript.

## Conflict of Interest Statement

The authors declare that the research was conducted in the absence of any commercial or financial relationships that could be construed as a potential conflict of interest.

## Footnotes

1 http://people.cas.sc.edu/rorden/mricron/main.html
