# Supplementary material for: Left Posterior Orbitofrontal Cortex Is Associated With Odor-Induced Autobiographical Memory: An fMRI Study
Source: Front Psychol. 2018 May 11;9:687. doi: 10.3389/fpsyg.2018.00687 (PMC5958215; doi:10.3389/fpsyg.2018.00687)
Supplement: Supplementary file 1 [file Data_Sheet_1.DOC]

SUPPLEMENTARY MATERIAL

**Left posterior orbitofrontal cortex is associated with**

**odor-induced autobiographical memory: an fMRI study**

Keiko Watanabe^1,2^, Yuri Masaoka^1*^, Mitsuru Kawamura^2^, Masaki Yoshida^3^, Nobuyoshi Koiwa^4^, Akira Yoshikawa^1^, Satomi Kubota^1,2^, Masahiro Ida^5^ , Kenjiro Ono^2^, Masahiko Izumizaki^1^

^1^Department of Physiology, Showa University School of Medicine, Tokyo, Japan

^2^Department of Neurology, Showa University School of Medicine, Tokyo, Japan

^3^Department of Ophthalmology, Jikei Medical University, Tokyo, Japan

^4^Human Arts and Sciences Research Center, University of Human Arts and Sciences, Saitama, Japan

^5^Department of Radiology, Stroke Center, Ebara Tokyo Hospital, Tokyo, Japan

*Corresponding author

Dr. Yuri Masaoka

Department of Physiology, Showa University School of Medicine

1-5-8 Hatanodai, Shinagawa-ku, Tokyo 142-8555, Japan

Telephone: +81 3 3784 8113; Fax: +81 3 3784 0200;

E-mail: [faustus@med.showa-u.ac.jp](mailto:faustus@med.showa-u.ac.jp)

**Supplementary Figure 1.**

Preservation of signal in basal frontal POFC. Example of the raw EPIs from a single subject (left) and a mask (right).

**Supplementary Figure 2.**

Exploratory whole brain results

Exploratory analyses across the whole brain did not reveal any additional regions exhibiting significant differences. Activations in other areas with thresholds of P < 0.05, P < 0.01 and P < 0.001 uncorrected for whole brain analysis for contrasts of *AM-odor>control* together with POFC activations were included as exploratory results in Supplementary Figure 1. Activations included right piriform cortex (x = 28, y = 14, z = −20), left piriform (x = −26, y = 10, z = −20), left brainstem (x = −6, y = −18, z = −20), right brainstem (x = 10, y = −18, z = −20), left fusiform gyrus (x = −44, y = −62, z = −20), left POFC (x = −36, y = 22, z = −10), right POFC (x = 35, y = 25, z = −10), right hippocampus (x = 22, y = −20, z = −14), left vmPFC (x = −10, y = 50, z = 0), right triangular part of the inferior frontal gyrus (x = 48, y = 32, z = −2), right thalamus proper (x = 10, y = −26, z = 0), left rdACC (x = −14, y = 36, z = 31), left superior frontal gyrus medial segment (x = −4, y = 32, z = 39) and right middle frontal gyrus (x = 34, y = −2, z = 34). POFC (x = −36, y = 22, z = −10, cluster size 251, SPMz = 3.53) activations surviving ROI analysis with FWE-adjusted statistical thresholds (P < 0.01) were obtained at cluster extent > 186.

**Supplementary Table 1:** **PPIs of the POFC and whole brain regions.**

**Supplementary Figure 3.**

Exploratory results of activations of primary olfactory and limbic regions from one subject

Activations in the primary olfactory regions and limbic areas at a threshold of P < 0.001 uncorrected (red) and P < 0.05 FWE corrected (yellow). Coordinates, brain regions, cluster size and z scores reached a threshold of P < 0.05 FWE corrected (yellow) are shown in Supplementary Table 1 below.

**Supplementary Table 2:** Primary olfactory cortex and limbic region activations

x, y, z = MNI coordinates at cluster peak, z = z scores.

Abbreviations: R, right; L, left; POFC, posterior orbitofrontal cortex; AMG, amygdala;

MSFC, middle superior frontal cortex; MFC, middle frontal cortex.

Regions reaching a statistical threshold of P < 0.05 FWE-corrected.
